# Longitudinal Mycobacterium tuberculosis-Specific Interferon Gamma Responses in Ethiopian HIV-Negative Women during Pregnancy and Postpartum

**DOI:** 10.1128/JCM.00868-21

**Published:** 2021-09-20

**Authors:** Fregenet Tesfaye, John Walles, Erik Sturegård, Niclas Winqvist, Taye Tolera Balcha, Mestawet Kefeni, Marianne Jansson, Per Björkman

**Affiliations:** a Clinical Infection Medicine, Department of Translational Medicine, Lund University, Malmö, Sweden; b Armauer Hansen Research Institute, Addis Ababa, Ethiopia; c Department of Infectious Diseases, Central Hospital, Kristianstad, Sweden; d Clinical Microbiology, Division of Laboratory Medicine, Lund University, Lund, Sweden; e Adama Public Health Research and Referral Laboratory Center, Adama, Ethiopia; f Medical Microbiology, Department of Laboratory Medicine, Lund University, Lund, Sweden; g Department of Infectious Diseases, Skåne University Hospital, Malmö, Sweden; Cepheid

**Keywords:** Ethiopia, interferon-γ, LTBI, pregnancy, QuantiFERON-TB Gold Plus

## Abstract

Pregnancy may influence cellular immune responses to Mycobacterium tuberculosis. We investigated M. tuberculosis-specific interferon-γ responses in women followed longitudinally during pregnancy and postpartum. Interferon-γ levels (stimulated by M. tuberculosis antigens [TB1 and TB2] and mitogen included in the QuantiFERON-TB Gold Plus assay) were measured in blood from pregnant HIV-negative women identified from a prospective cohort at Ethiopian antenatal care clinics. Longitudinal comparisons included women without active tuberculosis (TB) with M. tuberculosis-triggered interferon-γ responses of ≥ 0.20 IU/ml, sampled on two and/or three occasions (1st/2nd trimester, 3rd trimester, and 9 months postpartum). Among 2,093 women in the source cohort, 363 met inclusion criteria for longitudinal comparisons of M. tuberculosis-stimulated interferon-γ responses. Median M. tuberculosis-triggered interferon-γ concentrations were higher at 3rd than those at the 1st/2nd trimester (in 38 women with samples available from these time points; TB1: 2.8 versus 1.6 IU/ml, *P* = 0.005; TB2: 3.3 versus 2.8 IU/ml, *P* = 0.03) and postpartum (in 49 women with samples available from these time points; TB1: 3.1 versus 2.2 IU/ml, *P* = 0.01; TB2: 3.1 versus 2.3 IU/ml, *P* = 0.03). In contrast, mitogen-stimulated interferon-γ levels were lower at 3rd than those at 1st/2nd trimester (in 32 women with samples available from these time points: 21.0 versus 34.9 IU/ml, *P* = 0.02). Results were similar in 22 women sampled on all 3 occasions. In HIV-negative women, M. tuberculosis-stimulated interferon-γ responses were higher during the 3rd trimester than those at earlier stages of pregnancy and postpartum, despite decreased mitogen-triggered responses. These findings suggest increased M. tuberculosis-specific cellular responses due to dynamic changes of latent TB infection during pregnancy.

## INTRODUCTION

Tuberculosis (TB) is one of the leading nonobstetric causes of maternal morbidity and mortality, with the highest case burden in low-income countries (1, 2). Moreover, pregnancy has been associated with increased incidence of active TB (3, 4), probably related to physiological immunosuppression during pregnancy, which could lead to a loss of bacterial control in women with latent TB infection (LTBI). A high risk of active TB in connection to pregnancy has been reported in women with HIV (5, 6).

During pregnancy, the maternal immune system undergoes transient modification to prevent fetal rejection (7). These phenomena are most pronounced at later stages of pregnancy and are mainly characterized by suppression of T helper 1 (Th1) cell-mediated immunity and upregulation of regulatory T cells (Treg) (7–9).

Th1 cells are central for TB control and act by stimulating Mycobacterium tuberculosis-specific immunity and chemo-attraction of immune cells to sites of infection (10). Interferon-γ (IFN-γ) is a proinflammatory cytokine mainly produced by Th1 cells and has a key role in M. tuberculosis protective immunity (11). Pregnancy-related immune modulation influences overall IFN-γ secretion (8), an effect which could be particularly important for the control of TB infection.

Interferon-γ release assays (IGRAs) detect sensitization to M. tuberculosis by measuring plasma levels of IFN-γ after whole-blood incubation with M. tuberculosis specific antigens and have to a large extent replaced the tuberculin skin test (TST) for diagnostic testing for LTBI in high-income countries (12). Although the performance of IGRAs can be affected by conditions associated with reduced capacity for IFN-γ secretion, such as HIV infection and treatment with immunosuppressive drugs (13, 14), the potential influence of pregnancy on M. tuberculosis-triggered IFN-γ secretion has mainly been studied in women with HIV (15–17).

In a cross-sectional analysis of 829 participants in a cohort of Ethiopian pregnant women (among whom 5.9% were HIV-positive), we found that M. tuberculosis antigen-stimulated IFN-γ levels in the QuantiFERON-TB Gold Plus (QFT) assay were higher in women tested during the 3rd trimester than levels in those tested during the 1st or 2nd trimesters (18). In contrast, mitogen-induced IFN-γ levels were lower in women tested during the 3rd trimester (18).

To further explore the impact of pregnancy on M. tuberculosis-specific cellular immune responses, we compared M. tuberculosis-triggered IFN-γ levels in whole blood from HIV-negative women from the same cohort, sampled longitudinally during pregnancy and postpartum. In addition, we determined rates of conversion and reversion in QuantiFERON-TB Gold Plus reactivity between pregnancy and postpartum.

## MATERIALS AND METHODS

### Study participants and procedures.

Participants for this study were identified from a prospective cohort study on TB in women of reproductive age (19). Consenting individuals were included at their first antenatal care (ANC) visit during a current pregnancy at public health facilities in Adama, Ethiopia (November 2015 to February 2018). At inclusion and during follow-up, sociodemographic and medical information were collected, along with physical examination results. HIV testing was performed using rapid tests according to national guidelines (20). Mid-upper arm circumference (MUAC) was measured to assess malnutrition (using a cutoff level of 23 cm). Women with symptoms or signs suggestive of active TB (at inclusion or at any time during follow-up) were asked to submit two morning sputum samples for bacteriological TB analyses (smear microscopy, GeneXpert MTB/RIF PCR, and liquid culture). HIV-positive women were asked to submit such samples at inclusion, regardless of clinical manifestations. Active TB was defined as bacteriological detection of M. tuberculosis in sputum or fulfilment of clinical criteria for active TB in the absence of positive bacteriological results.

Follow-up visits were scheduled at up to three occasions during pregnancy and 9 months after delivery. Venous blood was collected in lithium-heparin tubes for QFT testing at inclusion and 9 months after delivery. From a subset of women, blood samples for QFT testing were also obtained at follow-up visits during the 3rd trimester (introduced August 2017; using the same procedure for blood collection).

For the present study, women with active TB (at inclusion or during follow-up) and a self-reported history of treatment for active TB and/or HIV infection were excluded.

### QFT testing.

After samples were transported to the study laboratory, 1 ml of heparinized whole blood was dispensed to each of four QFT incubation tubes (containing nil, mitogen, and TB1 and TB2 antigens) within 8 h of venipuncture. The tubes were mixed by inversion, incubated at 37°C for 18 h, and centrifuged. Aliquots of QFT plasma supernatants were stored at −20°C.

IFN-γ concentrations were measured using ELISA and analyzed using software according to the manufacturer’s protocol (Qiagen, Hilden, Germany) (21). IFN-γ levels after M. tuberculosis antigen and mitogen stimulation were calculated by subtracting background (nil) IFN-γ. Testing was repeated for samples with indeterminate results (mitogen-stimulated IFN-γ levels of <0.50 IU/ml and/or nil results of ≥8.0 IU/ml). Since our previous cross-sectional study showed lower mitogen-induced responses in women sampled at 3rd trimester than those in women sampled at 1st/2nd trimester, we also performed a 1:50 dilution of mitogen-stimulated samples with IFN-γ levels above the upper assay detection limit (10 IU/ml) to compare levels at these respective time points.

### Study definitions.

In order to improve detection of longitudinal fluctuations in IFN-γ secretion and to account for the potentially reduced capacity of IFN-γ secretion during pregnancy (17), we used a cutoff level of 0.20 IU/ml (instead of the standard cutoff level of 0.35 IU/ml) as the criterion for inclusion. Women with M. tuberculosis-triggered IFN-γ of ≥0.20 IU/ml in either of the TB antigen tubes on all sampling occasions were included for the comparison of M. tuberculosis-triggered IFN-γ levels during pregnancy and postpartum. Data for women sampled at 1st and 2nd trimester were combined since the main focus of this analysis concerned changes in IFN-γ secretion at late stages of pregnancy.

For the analysis of QFT conversions and reversions, women with valid QFT results during pregnancy and postpartum were considered for inclusion. Participants with M. tuberculosis-triggered IFN-γ levels within a borderline zone (defined as 0.20 to 0.70 IU/ml [22]) were excluded from this analysis. QFT conversion was defined as an initial M. tuberculosis-triggered IFN-γ level of <0.20 IU/ml (in both antigen preparations), with a follow-up result of ≥0.70 IU/ml (in one or both of the antigen preparations). QFT reversion was defined as an initial M. tuberculosis-triggered IFN-γ level of ≥0.70 IU/ml (in one or both of the antigen preparations) with a follow-up result of <0.20 IU/ml (in both antigen preparations).

### Statistical analysis.

Pairwise longitudinal comparisons of M. tuberculosis-triggered IFN-γ levels were performed between 1st/2nd versus 3rd trimester, 1st/2nd versus postpartum, and 3rd trimester versus postpartum separately for the two antigen preparations using the Wilcoxon signed-rank test. Mitogen-induced IFN-γ responses were compared between 1st/2nd and 3rd trimesters with this test. A longitudinal comparison of M. tuberculosis-antigen stimulated IFN-γ responses was also performed using the Friedman test, followed by Dunn’s multiple comparisons for participants serially tested at all three time points (1st/2nd trimester, 3rd trimester, and postpartum).

QFT conversions and reversions were analyzed between samples obtained during pregnancy (irrespective of trimester) and postpartum. Results were presented with medians.

*P* values of <0.05 were considered significant. All statistical analyses were performed using SPSS statistics 25 and GraphPad prism version 8.4.2.

### Ethical statement.

This study was approved by the National Research Ethics Review Committee, Addis Ababa, Ethiopia, and the Regional Ethical Review Board, Lund University, Sweden. Written informed consent was obtained from participants prior to enrollment.

## RESULTS

### Participants.

**FIG 1:**
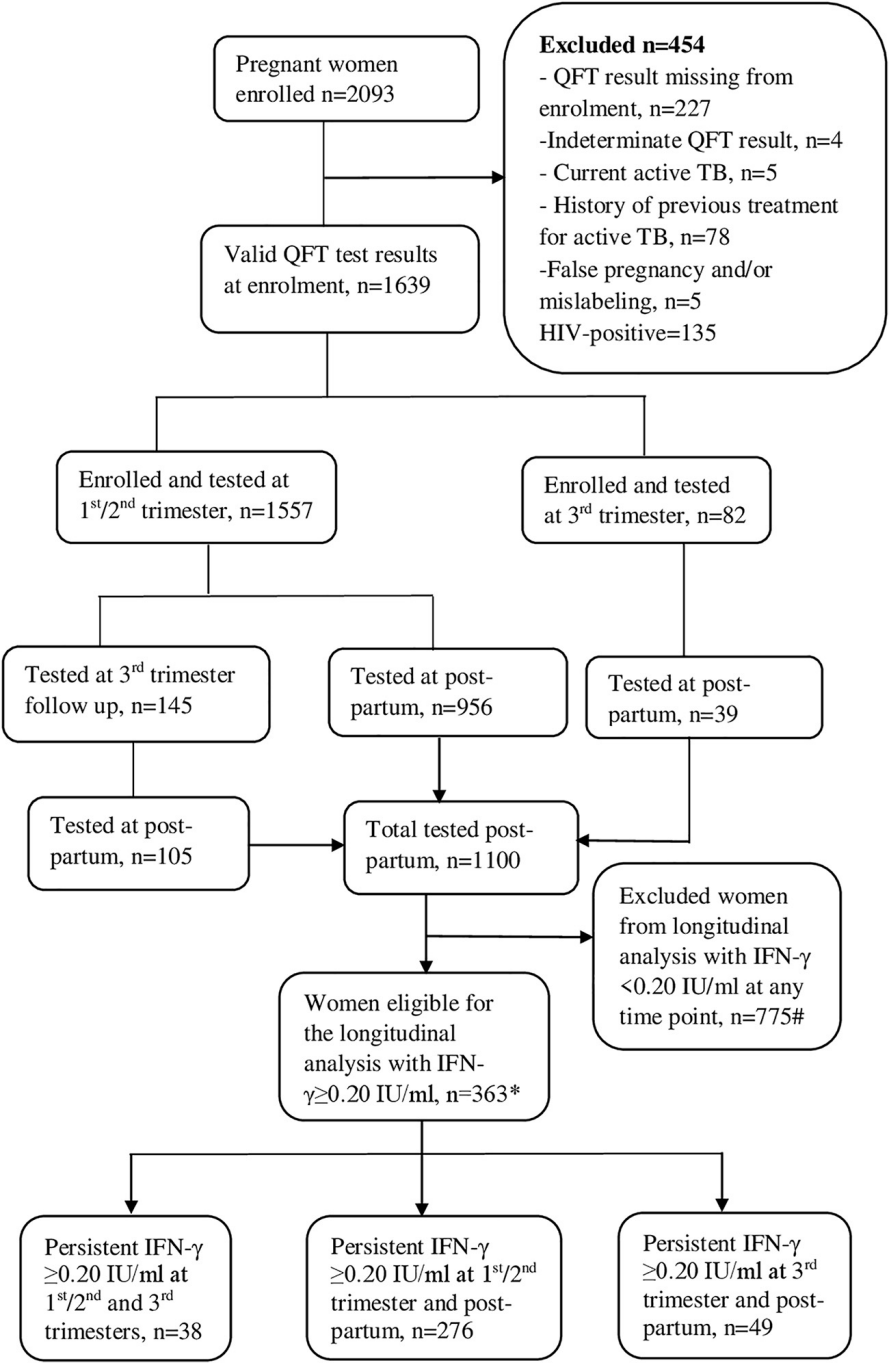
Flow chart of pregnant women followed during pregnancy and postpartum that were included for an analysis of longitudinal M. tuberculosis-triggered IFN-γ responses. #, includes 16 women tested at different time points; *, includes 22 women with samples available from 1st/2nd trimester, 3rd trimester, and postpartum.

Among 2,093 pregnant women enrolled in the source cohort, 1,639 had valid QFT results at inclusion; 454 were excluded for different reasons (Fig. 1). Of 1,639 pregnant women, 1,557 (95%) were enrolled during the 1st or 2nd trimester (1st trimester 375 [24%] versus 2nd trimester 1,182 [76%]), whereas 82 (5%) women were enrolled during the 3rd trimester. The median age at inclusion was 25 years (interquartile range [IQR], 22 to 27), with a median gestational age of 18 weeks (IQR, 14 to 21). One-hundred forty-five women included during the 1st/2nd trimester were retested during the 3rd trimester. QFT results were available from 1,100 participants at ≥9 months postpartum (Fig. 1). Three-hundred sixty-three women were sampled on two or three occasions, had persistent QFT IFN-γ responses of ≥0.20 IU/ml, and were included in the longitudinal analysis of M. tuberculosis-triggered IFN-γ secretion (Fig. 1). Baseline characteristics of women included in this analysis are shown in Table S1 in the supplemental material.

For an analysis of patterns of QFT conversion and reversion between pregnancy and postpartum, 610 women with QFT results of <0.20 IU/ml during pregnancy and 283 women with QFT results of >0.70 IU/ml during pregnancy were included (see Fig. S3 in the supplemental material). Baseline characteristics of women included in this analysis are shown in Table S4 in the supplemental material.

### Comparison of M. tuberculosis-triggered IFN-γ responses at different time points during pregnancy and postpartum.

**FIG 2:**
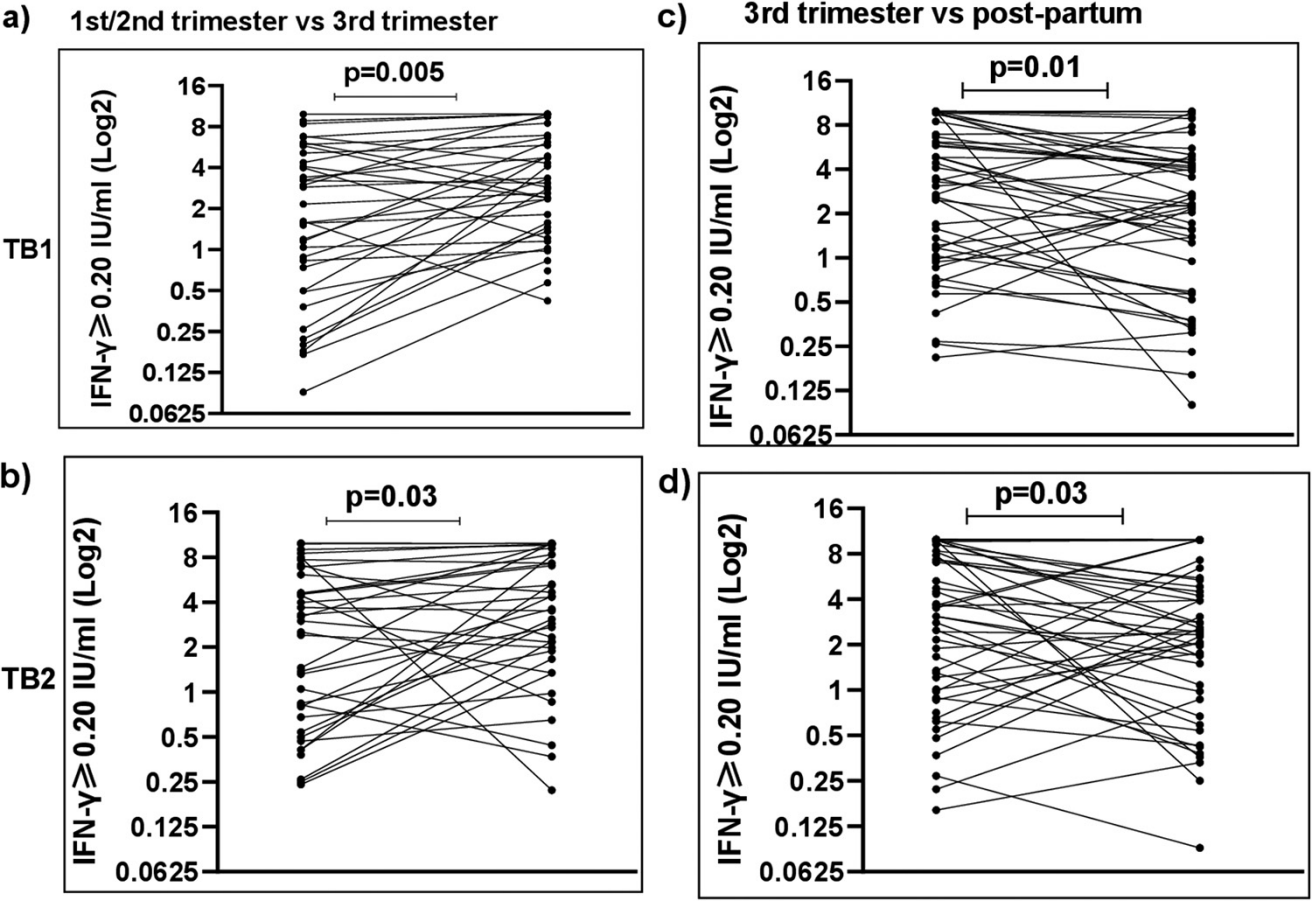
M. tuberculosis-antigen (TB1 and TB2) stimulated IFN-γ levels (≥0.20 IU/ml) in women at 1st/2nd trimester, 3rd trimester, and postpartum. The Wilcoxon signed-rank test was used to calculate IFN-γ level differences between 1st/2nd trimester and 3rd trimester (*n* = 38) (a and b) and between 3rd trimester and postpartum (*n* = 49) (c and d).

In order to study the dynamics of M. tuberculosis-triggered responses during pregnancy and postpartum, we longitudinally compared IFN-γ levels stimulated by TB1 and TB2 antigens in blood obtained at the following time points: 1st/2nd trimester, 3rd trimester, and postpartum. We initially performed pairwise comparisons for women with results from these time points (Fig. 2 and Fig. S1). The distributions of QFT IFN-γ levels according to different categories at these time points are summarized in Table S2 in the supplemental material. No statistically significant differences were observed by comparing women sampled at the 1st and 2nd trimester (data not shown).

Among 363 women with an IFN-γ of ≥0.20 IU/ml, 38 (10.5%) women were sampled both at the 1st/2nd and 3rd trimester. Median M. tuberculosis-stimulated IFN-γ concentrations were greater at the 3rd than those at the 1st/2nd trimester (TB1: 2.8 versus 1.6 IU/ml, *P* = 0.005; TB2: 3.3 versus 2.8 IU/ml, *P* = 0.03) (Fig. 2a and b). Mitogen-stimulated IFN-γ concentrations were >10 IU/ml in 28/38 and 22/38 participants tested at the 1st/2nd and 3rd trimesters, respectively. After dilution (samples from 32/38 of these women), mitogen-stimulated IFN-γ levels were found to be lower at the 3rd than those at the 1st/2nd trimester (21.0 versus 34.9 IU/ml, *P* = 0.02) (see Fig. S2 in the supplemental material).

Two-hundred seventy-six (76%) women were tested at the 1st/2nd trimester and postpartum. Median levels of M. tuberculosis-triggered IFN-γ were similar postpartum compared with 1st/2nd trimester (TB1: 2.3 versus 1.9 IU/ml, *P* = 0.60; TB2: 2.5 versus 1.9 IU/ml, *P* = 0.20) (Fig. S1).

Forty-nine (13.5%) women were sampled at the 3rd trimester and postpartum. M. tuberculosis-stimulated IFN-γ levels were greater at the 3rd trimester than those postpartum (TB1: 3.1 versus 2.2 IU/ml, *P* = 0.01; TB2: 3.1 versus 2.3 IU/ml, *P* = 0.03) (Fig. 2c and d).

### Longitudinal M. tuberculosis-triggered IFN-γ levels at 1st/2nd trimester, 3rd trimester, and postpartum.

**FIG 3:**
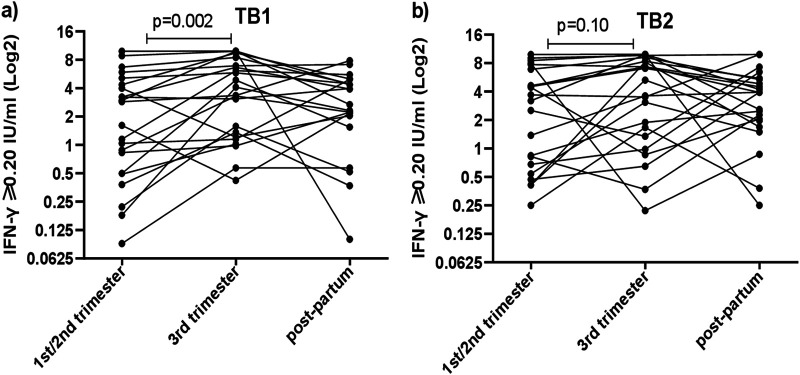
The line plots represent changes in IFN-γ concentrations triggered by TB1 (a) and TB2 (b) in whole blood of 22 longitudinally tested women (all with QFT of ≥0.20 IU/ml), followed during pregnancy and postpartum. The Friedman test was used to analyze the median difference in the three-time point longitudinal series.

From 22 women, samples were available from all 3 occasions (Fig. 3). Median TB1-stimulated IFN-γ levels were significantly higher at 3rd trimester than those at 1st/2nd trimester (3.7 versus 2.2 IU/ml, *P* = 0.002), whereas these levels were not statistically different at 3rd trimester compared with those at postpartum (3.7 vs 2.5 IU/ml, p=0.20) nor at 1st/2nd trimester compared with post-partum (2.2 versus 2.5 IU/ml; p=0.30) (Fig. 3a).

In TB2-stimulated samples, no significant difference was found for median IFN-γ levels at 3rd compared with the 1st/2nd trimester (4.5 vs 3.4 IU/ml, p=0.10), nor between postpartum and 1st/2nd trimester (3.9 versus 3.4 IU/ml, *P* = 0.6), nor between 3rd trimester and postpartum (4.5 versus 3.9 IU/ml, *P* = 0.90) (Fig. 3b).

### QFT conversions and reversions between pregnancy and postpartum.

In 71/610 (11.6%) women with QFT of <0.20 IU/ml during pregnancy, postpartum M. tuberculosis-triggered IFN-γ levels were ≥0.70 IU/ml (median IFN-γ levels of 1.13 versus 0.01 IU/ml [TB1] and 1.72 versus 0.01 IU/ml [TB2]), which meets our definition of QFT conversion.

Among 283 women with QFT results of ≥0.70 IU/ml during pregnancy, 52 (18.4%) showed IFN-γ responses of <0.20 IU/ml postpartum (median IFN-γ levels of 1.5 versus 0.00 IU/ml [TB1] and 2.2 versus −0.01 IU/ml [TB2]), thus meeting our definition for QFT reversion.

## DISCUSSION

In this study of longitudinal M. tuberculosis-triggered IFN-γ responses in HIV-negative women followed during pregnancy and postpartum, we found that M. tuberculosis-stimulated IFN-γ levels were higher during the 3rd trimester than those during both postpartum and earlier stages of pregnancy, which is in contrast to mitogen-triggered IFN-γ secretion, which was lower at later than earlier stages of pregnancy.

Protective M. tuberculosis immunity is predominantly based on Th1 cellular responses, which trigger the secretion of proinflammatory cytokines, such as IFN-γ. During pregnancy, Th1 cellular responses are downregulated, with a corresponding increase in regulatory T cells (8, 23). These immune modulations have been linked to reactivation of latent infections and elevated susceptibility for a range of infectious diseases, such as malaria, and those mediated by cytomegalovirus and other herpesviruses (23, 24). These phenomena might also be involved in the increased incidence of active TB during pregnancy and early-postpartum period, which has been reported in registry-based studies (3, 25, 26).

Apart from increasing the risk of LTBI reactivation, pregnancy may also affect the performance of immune-based diagnostic tests. Several studies report lower detection rates using TST than using a prior generation IGRA test, QuantiFERON-TB Gold In Tube (QFT-GIT), in pregnant women (16, 27). Published findings on the impact of pregnancy on M. tuberculosis-triggered IFN-γ secretion are partly contradictory. In a cross-sectional study of HIV-negative women in New York City, NY, rates of QFT-GIT positivity were similar in women tested at different trimesters, with comparable results observed in nonpregnant women, and no significant differences in IFN-γ levels in a subset of 25 women retested during pregnancy or postpartum (28). In contrast, M. tuberculosis-triggered IFN-γ concentrations were lower in samples obtained at delivery than those antepartum or postpartum in a cross-sectional study of HIV-negative Indian women, with similar trends in 60 women followed longitudinally (27). To our knowledge, other longitudinal studies of M. tuberculosis-triggered IFN-γ secretion in connection to pregnancy have exclusively targeted HIV-positive women. In these studies, IFN-γ responses were found to be decreased during pregnancy, with subsequent higher responses in samples obtained after delivery (15, 16, 29). Our finding of increased M. tuberculosis-specific responses during the 3rd trimester are in clear contrast to those recently reported by Weinberg et al. (15). In their large multicenter study comparing immediate and deferred isoniazid preventive therapy in pregnant women with HIV, a longitudinal analysis of M. tuberculosis-triggered IFN-γ secretion showed a significant reduction at later stages of pregnancy compared with early pregnancy and postpartum (15).

In this study, we compared IFN-γ secretion in women sampled on at least two occasions in relation to pregnancy. M. tuberculosis-triggered responses were higher during the 3rd trimester, whereas no differences were observed between 1st/2nd trimester and postpartum, implying that these changes occur during later stages of pregnancy. Although our findings on longitudinal M. tuberculosis-triggered IFN-γ secretion were similar for TB1 and TB2 antigen stimulation, the differences in kinetics were less pronounced for TB2 than TB1, potentially accounting for by the different mixes of TB antigens in these two stimulations. The TB2 tube included in the QFT-Plus assay contains both long and short peptide antigens designed to stimulate CD4 and CD8 T cell responses, respectively, which has been associated with a better capacity to induce IFN-γ secretion in immunosuppressed hosts and in recently infected persons (30, 31).

Our finding of elevated M. tuberculosis-triggered IFN-γ secretion during the 3rd trimester suggests an increased stimulation of M. tuberculosis-specific T cells at later stages of pregnancy, despite lower overall T-cell responsiveness, as reflected by decreased mitogen-triggered IFN-γ levels. However, with the current study design, we were not able to investigate the mechanisms responsible for this phenomenon. We speculate that this phenomenon might be related to reduced control of bacterial replication in women with LTBI and could represent increased internal exposure to M. tuberculosis antigens. Although known contact with active TB was rarely reported among participants, we cannot exclude that repeated TB exposure between these time points could contribute to this pattern. In our study population, we observed individual variations in longitudinal M. tuberculosis-triggered IFN-γ responses; although most participants showed increased reactivity at the 3rd trimester, a subset of women displayed reductions of these responses during later stages of pregnancy. This discrepancy could be explained by spontaneous bacterial clearance that can occur in some individuals with immunological signs of LTBI, and the dampened M. tuberculosis-triggered IFN-γ secretion might reflect an inhibition of TB-specific memory T cell responses, similar to the reduced mitogen responses occurring in late pregnancy (8, 24, 25).

It is likely that the discordant findings with regard to M. tuberculosis-triggered IFN-γ secretion during the 3rd trimester between our and other longitudinal studies performed in women with HIV reflect differences in a woman’s capacity to mount cellular responses to such increased M. tuberculosis antigen exposure. Probably, most pregnant HIV-negative women with LTBI manage to control their TB infection, despite the physiological immune modification that occurs during pregnancy. Taken together with previously reported data, these findings imply a profound effect of HIV infection on the immune control of LTBI in connection with pregnancy, which is likely to be involved in the increased incidence of pregnancy-associated active TB in women with HIV. Pregnant women with HIV living in regions of TB endemicity are at high risk of active TB despite receipt of antiretroviral therapy (ART) and relatively normal CD4 cell counts (32).

We used IFN-γ as a marker of Th1-cell responsiveness; an analysis of a broader range of immune mediators might have provided more detailed information on how pregnancy affects the immune control of LTBI in relation to pregnancy. For example, Mathad et al. reported decreasing levels of both IFN-γ and IL-2 during pregnancy in HIV-positive women who subsequently developed active TB (17). In a large Norwegian register-based study of persons screened with QFT for assessment of LTBI (in which 2% of participants were HIV positive), higher M. tuberculosis-stimulated IFN-γ levels were associated with the risk of incident TB (33). The increased IFN-γ responses that we observed during the 3rd trimester might represent transient changes in the TB infection spectrum from truly latent to incipient active TB. However, a follow-up of participants in this study has so far not revealed any cases of active TB.

Since we previously observed that a relatively high proportion of pregnant women had M. tuberculosis-triggered IFN-γ levels within the lower borderline zone (0.20 to 0.35 IU/ml) and to allow for detection of variations in IFN-γ secretion during the course of pregnancy, we chose this lower cutoff to select participants for this longitudinal analysis. Similar results were found using 0.35 IU/ml as the cutoff level (see Table S3 in the supplemental material). Our data suggest that the recommended cutoff for the definition of QFT positivity (0.35 IU/ml) is adequate in pregnancy, at least for HIV-negative women.

For this study, we also analyzed the incidence of conversions and reversions in QFT reactivity. Since IGRAs are subject to variability around the threshold level (0.35 IU/ml), we chose cutoff levels at the lower and upper limits of the commonly used borderline zone (0.20 to 0.70 IU/ml) (22). In this study, we observed a high QFT conversion rate (11.6%) in women tested during pregnancy and postpartum. Although the reduced capacity for IFN-γ secretion during pregnancy could be considered a potential mechanism for QFT conversion, our finding of elevated levels of M. tuberculosis-triggered responses during the 3rd trimester, as well as similar IFN-γ levels during 1st/2nd trimester and postpartum, do not support this hypothesis. We consider it more likely that these conversions represent newly acquired TB infections, which is in line with recently presented findings from this cohort, showing a strong association with increasing age and LTBI prevalence and suggesting continuous unrecognized exposure to contagious TB among women in our uptake area (19). This finding emphasizes the need for improved control of active TB case finding in areas of TB endemicity.

Besides conversion, we observed QFT reversions in 52 women (18.4%), which is a relatively high rate compared with other longitudinal studies performed in connection to pregnancy (27, 28). The reasons for this phenomenon are not apparent. In high-income countries, QFT reversions (commonly around the cutoff level of 0.35 IU/ml) are often attributed to test variability (34). QFT reversion could also be due to weaning immune responses to M. tuberculosis antigens, indicating spontaneous clearance of TB infection (35). In settings with ongoing TB transmission, however, reversion of QFT reactivity in persons with recently acquired TB infection has been linked to an increased risk of developing active TB, as shown in a large prospective study of South African adolescents with repeated QFT testing and follow-up for 5 years (36). Thus far, we have not observed incident active TB among these 52 women.

In the current study, we evaluated IFN-γ responses in HIV-negative women living in an area of TB endemicity, who were identified from a large prospective cohort. Participants were sampled and retested during follow-up regardless of initial QFT results. Furthermore, participants were followed to assess the incidence of active TB, with bacteriological testing in clinically suspected cases. To our knowledge, this is the largest cohort study with a longitudinal assessment of M. tuberculosis-specific IFN-γ responses in HIV-negative women and the first to use the QFT-Plus assay.

We also acknowledge certain limitations. Only a minority of women were sampled on all three occasions; however, findings in this subset were similar to those with samples restricted to two time points. A comparison of patterns of M. tuberculosis-triggered IFN-γ secretion in women with HIV from the same uptake area would have been of interest with regard to previous data in such women from other settings. However, few women with HIV in our cohort had available 3rd trimester samples. Furthermore, women with HIV is a heterogeneous group with regard to factors that affect M. tuberculosis-induced Th1 responses (especially degree of immunosuppression and receipt of antiretroviral therapy), and adjusting for these factors is necessary for a representative comparison. For these reasons, we chose to restrict our study to HIV-negative participants from the source cohort.

Apart from HIV infection, other comorbidities, for which we did not have information, could affect Th1 cell responsiveness. For example, parasitic and helminth infections have been shown to increase the occurrence of indeterminate QFT results (37). A final limitation, similar to all human studies of LTBI, is the lack of a test that can distinguish persons with positive QFT results with persistent viable bacilli from those with spontaneously resolved infection and remaining immunological memory.

In conclusion, M. tuberculosis-triggered IFN-γ responses showed dynamic changes during pregnancy and postpartum in this cohort of Ethiopian HIV-negative women. In contrast to studies restricted to HIV-positive women, we found increased IFN-γ responses in the 3rd trimester compared with those in earlier stages of pregnancy and postpartum. This finding suggests higher M. tuberculosis antigen exposure, which could be due to transiently increased bacterial replication in latently infected women during pregnancy.
