# Supplementary material for: Longitudinal Mycobacterium tuberculosis-Specific Interferon Gamma Responses in Ethiopian HIV-Negative Women during Pregnancy and Postpartum
Source: J Clin Microbiol. 2021 Sep 20;59(10):e00868-21. doi: 10.1128/JCM.00868-21 (PMC8451422; doi:10.1128/JCM.00868-21)
Supplement: Supplemental file 1 — Tables S1 to S4 and Fig. S1 to S4. Download JCM.00868-21-s0001.pdf, PDF file, 0.4 MB [file jcm.00868-21-s0001.pdf]

Supplementary Data

Supplementary Table 1. Baseline characteristics of 363 women included for analysis of longitudinal Mtb-triggered IFN-γ responses

| Characteristics* | Total | 1st/2nd vs 3rd trimenster (n; %) | 3rd trimester vs postpartum (n; %) | 1st/2nd trimester vs post-partum (n; %) |
|---|---|---|---|---|
| Total | 363 | 38 | 49 | 276 |
| **Age (years)** | | | | |
| ≤20 | 46 | 10 (26.3) | 8 (16.3) | 28 (10.1) |
| 21-25 | 122 | 7 (18.4) | 14 (28.5) | 101 (36.6) |
| 26-30 | 155 | 16 (42.1) | 22 (45) | 117 (42.4) |
| ≥31 | 39 | 5 (13.2) | 5 (10.2) | 29 (10.5) |
| **Marital status** | | | | |
| Single | 11 | 1 (2.6) | 2 (4) | 8 (2.9) |
| Married | 351 | 37 (97.4) | 47 (96) | 267 (96.7) |
| **Parity** | | | | |
| First pregnancy | 104 | 8 (21.1) | 14 (28.6) | 82 (29.7) |
| Previous pregnancies | 255 | 29 (76.3) | 35 (71.4) | 191 (69.2) |
| **MUAC (cm)** | | | | |
| ≤23 | 93 | 7 (18.4) | 9 (18.4) | 77 (28) |
| >23 | 268 | 31 (81.6) | 40 (81.6) | 197 (71.3) |

Footnotes

*Missing data n (%) for parity 1 (2.6) at 1$^{st}$/2$^{nd}$ vs 3$^{rd}$ trimester and for age 1 (0.4), marital status 1 (0.4), parity 3 (1.1) and MUAC 2 (0.7) at 1$^{st}$/2$^{nd}$ trimester vs post-partum.

MUAC**:** mid upper arm circumference

**Supplementary Table 2.** Distribution of women according to QFT IFN-γ category at different time points during pregnancy and post-partum

| | Study Visits in the cohort | | |
|---|---|---|---|
| **QFT IFN-γ categories** | **1st/2nd trimester** | **3rd trimester** | **Postpartum** |
| Total | 1557 | 227 | 1100 |
| | N (%) | N (%) | N (%) |
| ≥0.20 IU/ml | 577 (37.1) | 96 (42.3) | 471 (42.8) |
| TB1+/TB2+ | 487 (31.3) | 78 (34.4) | 360 (32.7) |
| TB1+/TB2- | 34 (2.2) | 6 (2.6) | 38 (3.5) |
| TB1-/TB2+ | 56 (3.6) | 12 (5.3) | 73 (6.6) |
| <0.20 IU/ml | | | |
| TB1-/TB2- | 980 (62.9) | 131 (57.7) | 629 (57.2) |
| 0.20–0.70 IU/ml[a] | | | |
| TB1 and/ TB2 | 187 (12) | 24 (10.6) | 159 (14.5) |
| 0.20–0.34 IU/ml[b] | | | |
| TB1 and/TB2 | 86 (5.5) | 14 (6.2) | 70 (6.4) |
| 0.35–0.70 IU/ml[c] | | | |
| TB1 and/TB2 | 122 (7.8) | 13 (5.7) | 97 (8.8) |
| ≥0.70 IU/ml | | | |
| TB1 and/TB2 | 431 (27.7) | 78 (34.4) | 345 (31.4) |

Footnotes

[a]All borderline results (in TB1 and/or TB2 antigen)

[b]Low borderline (in TB1 and/or TB2 antigen)

[c]High borderline (in TB1 and/or TB2 antigen)

**Supplementary Table 3**. Comparison of Mtb-triggered IFN-γ responses at different time points during pregnancy and post-partum using the recommended cut-off point (IFN-γ ≥0.35 IU/ml)

| | **1st/2nd vs 3rd trimester¤** | **P value** | **1st/2nd trimester vs post-partum¤** | **P value** | **3rd trimester vs post-partum¤** | **P value** | **1st/2nd vs 3rd trimeter vs Post-partum#** | **P value** |
|---|---|---|---|---|---|---|---|---|
| N (total=336) | 35 | | 235 | | 46 | | 20 | |
| TB1 (IFN-γ≥0.35 IU/ml)* | 2.2 (0.8–5) vs 3.1 (1.2–6) | 0.01 | 2.5 (0.9–6) vs 2.7 (1.1–5.4) | 0.8 | 3.2 (1–6.3) vs 2.3 (0.9–4.5) | 0.01 | 2.2 (0.58–5) vs 3.7 (1.2–6.9) vs 3.3 (2.1–5.5) | 0.006 |
| TB2 (IFN-γ≥0.35 IU/ml)* | 3.2 (0.8–6) vs 3.6 (1.2–8.3) | 0.08 | 2.5 (1.1–7) vs 2.9 (1.4–6.5) | 0.3 | 3.3 (1–8) vs 2.5 (1–5) | 0.05 | 4.1 (0.7–7.5) vs 4.5 (1.1–8.3) vs 3.9 (2–5.5) | 0.3 |
| N (total=363) | 38 | | 276 | | 49 | | 22 | |
| TB1 (IFN-γ ≥0.20 IU/ml)* | 1.6 (0.5–4.5) vs 2.8 (1.3–6) | 0.005 | 1.9 (0.5–5) vs 2.3 (0.8–5) | 0.6 | 3.1 (1–6.4) vs 2.2 (0.6–4.5) | 0.01 | 2.2 (0.5–4.6) vs 3.7 (1.2–7.3) vs 2.5 (1.3–4.6) | 0.002 |
| TB2 (IFN-γ≥0.20 IU/ml)* | 2.8 (0.6–5) vs 3.3 (1.3–7.5) | 0.03 | 1.9 (0.7–5.4) vs 2.5 (0.9–5.7) | 0.2 | 3.1(0.9–8) vs 2.3 (0.6–4.7) | 0.03 | 3.4 (0.6–7) vs 4.5 (1.3–8.6) vs 3.9 (1.9–5.4) | 0.1 |

Footnotes

*Median (interquartile range; IQR) IFN-γ longitudinal response in participants with persistent QFT results ≥0.35 IU/ml and ≥0.20 IU/ml in TB1 and/or TB2 antigens

¤Wilcoxon matched pairs signed rank test was performed for pairwise comparisons.

#Friedman test, followed by Dunn's multiple comparisons was used for participants serially tested at three time points and the p values indicated for the comparison between $1^{st}/2^{nd}$ vs $3^{rd}$ trimester.

**Supplementary Table 4.** Baseline characteristics of women included for analysis of QFT conversions (n=610) and reversions (n=283) between pregnancy and post-partum.

| | **QFT IFN-γ <0.20 IU/ml during pregnancy** | | | **QFT IFN-γ >0.70 IU/ml during pregnancy** | | |
|---|---|---|---|---|---|---|
| Characteristics* | Total | Converters (n; %) | Non-converters (n; %) | Total | Reverters (n; %) | Non-Reverters (n; %) |
| Total | 610 | 71 | 539 | 283 | 52 | 231 |
| **Age (years)** | | | | | | |
| ≤20 | 123 | 12 (17) | 111 (20.6) | 41 | 11 (21.2) | 30 (13) |
| 21-25 | 266 | 32 (45) | 234 (43.4) | 107 | 24 (46.2) | 83 (36) |
| 26-30 | 185 | 22 (31) | 163 (30.2) | 105 | 10 (19.2) | 95 (41.1) |
| ≥31 | 34 | 4 (5.6) | 30 (5.6) | 29 | 7 (13.4) | 22 (9.5) |
| **Marital status** | | | | | | |
| Single | 17 | 3 (4.2) | 14 (2.6) | 7 | 0 | 7 (3) |
| Married | 584 | 67 (94.4) | 517 (96) | 275 | 52 (100) | 223 (96.5) |
| Divorced | 6 | 0 | 6 (1) | 0 | 0 | 0 |
| Widowed | 1 | 1 (1.4) | 0 | 0 | 0 | 0 |
| **Parity** | | | | | | |
| First pregnancy | 239 | 33 (46.5) | 206 (38.2) | 95 | 23 (44.2) | 72 (31.2) |
| Previous pregnancies | 345 | 35 (49.3) | 310 (57.5) | 183 | 26 (50) | 157 (68) |
| **MUAC (cm)** | | | | | | |
| ≤23 | 206 | 28 (39.4) | 178 (33) | 79 | 18 (34.6) | 61 (26.4) |
| >23 | 395 | 42 (59.2) | 353 (65.5) | 201 | 33 (63.4) | 168 (72.7) |

*Missing data n (%) for age, marital status, parity and MUAC:- converters: 1 (1.4), 0 (0), 3 (4.2) and 1 (1.4); non-convertors: 1 (0.2), 2 (0.4), 23 (4.3) and 8 (1.5); reverters: 0 (0), 0 (0), 3 (5.8) and 1 (2); non-reverters: 1 (0.4), 1 (0.4), 2 (0.8) and 2 (0.9), respectively.

MUAC**:** mid upper arm circumference

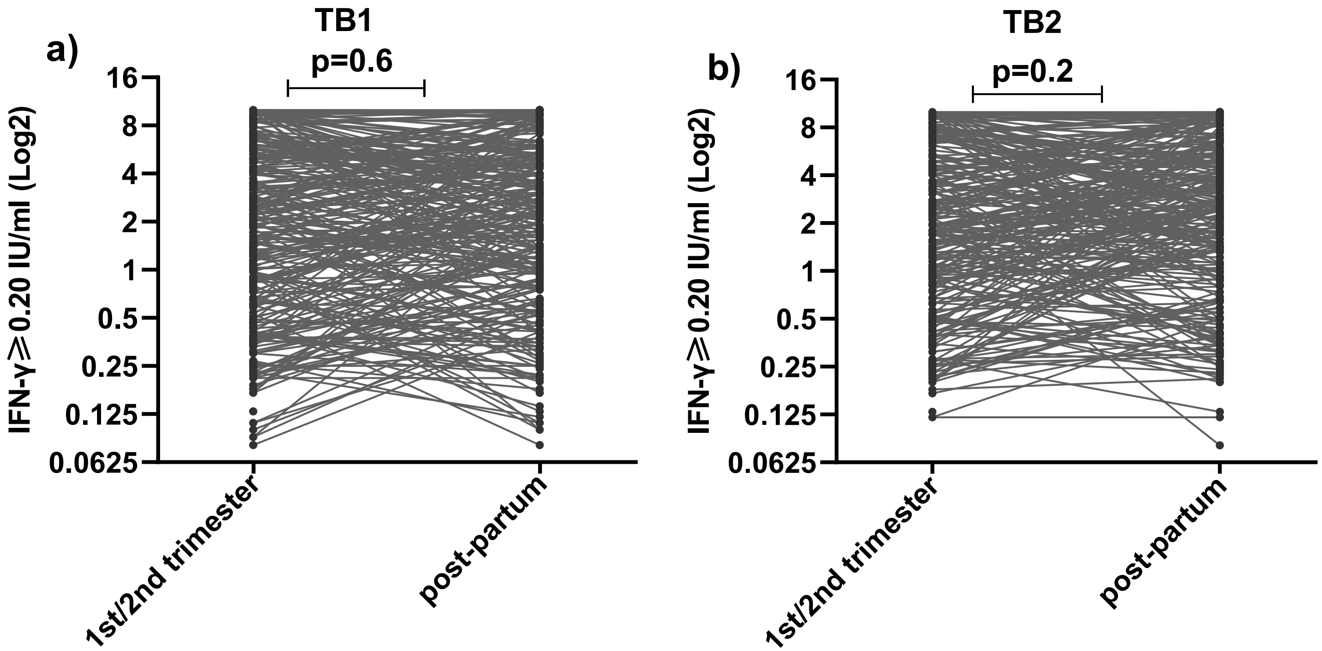
a)
TB1
p=0.6
IFN-γ≥0.20 IU/ml (Log2)
16
8
4
2
1
0.5
0.25
0.125
0.0625
1st/2nd trimester
post-partum
b)
TB2
p=0.2
IFN-γ≥0.20 IU/ml (Log2)
16
8
4
2
1
0.5
0.25
0.125
0.0625
1st/2nd trimester
post-partum

**Supplementary Figure 1**. Mtb-antigen (TB1(a) and TB2 (b)) stimulated IFN-γ levels (≥0.20 IU/ml) in women at $1^{st}/2^{nd}$ trimester and post-partum (n=276). Wilcoxon matched-pairs signed rank test was used to calculate IFN-γ level difference between these time points.

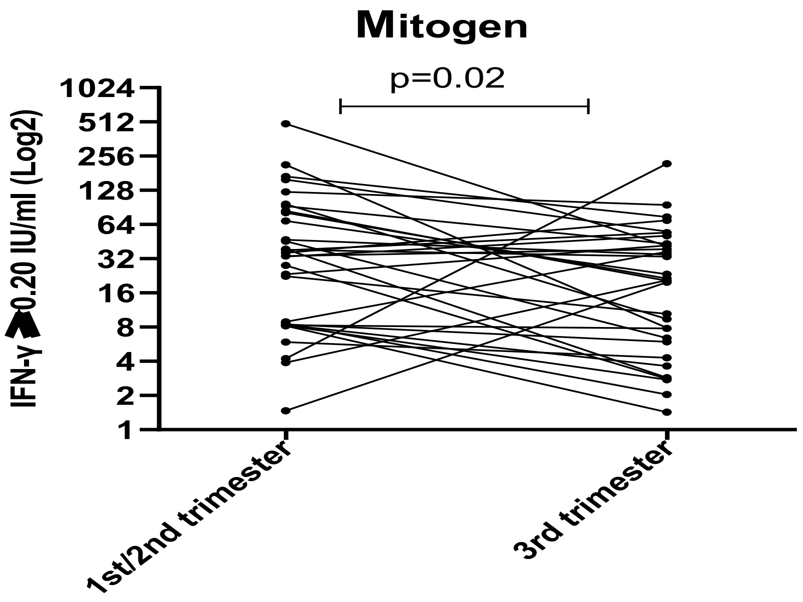
Mitogen
p=0.02
IFN-γ ≥0.20 IU/ml (Log2)
1024
512
256
128
64
32
16
8
4
2
1
1st/2nd trimester
3rd trimester

**Supplementary Figure 2**. Mitogen-stimulated IFN-γ longitudinal response after dilution in 32/38 women tested at $1^{st}/2^{nd}$ and $3^{rd}$ trimester with median IFN-γ levels 34.9 vs 21.0 IU/ml, respectively. Wilcoxon matched-pairs signed rank test was used to calculate IFN-γ level difference between these time points

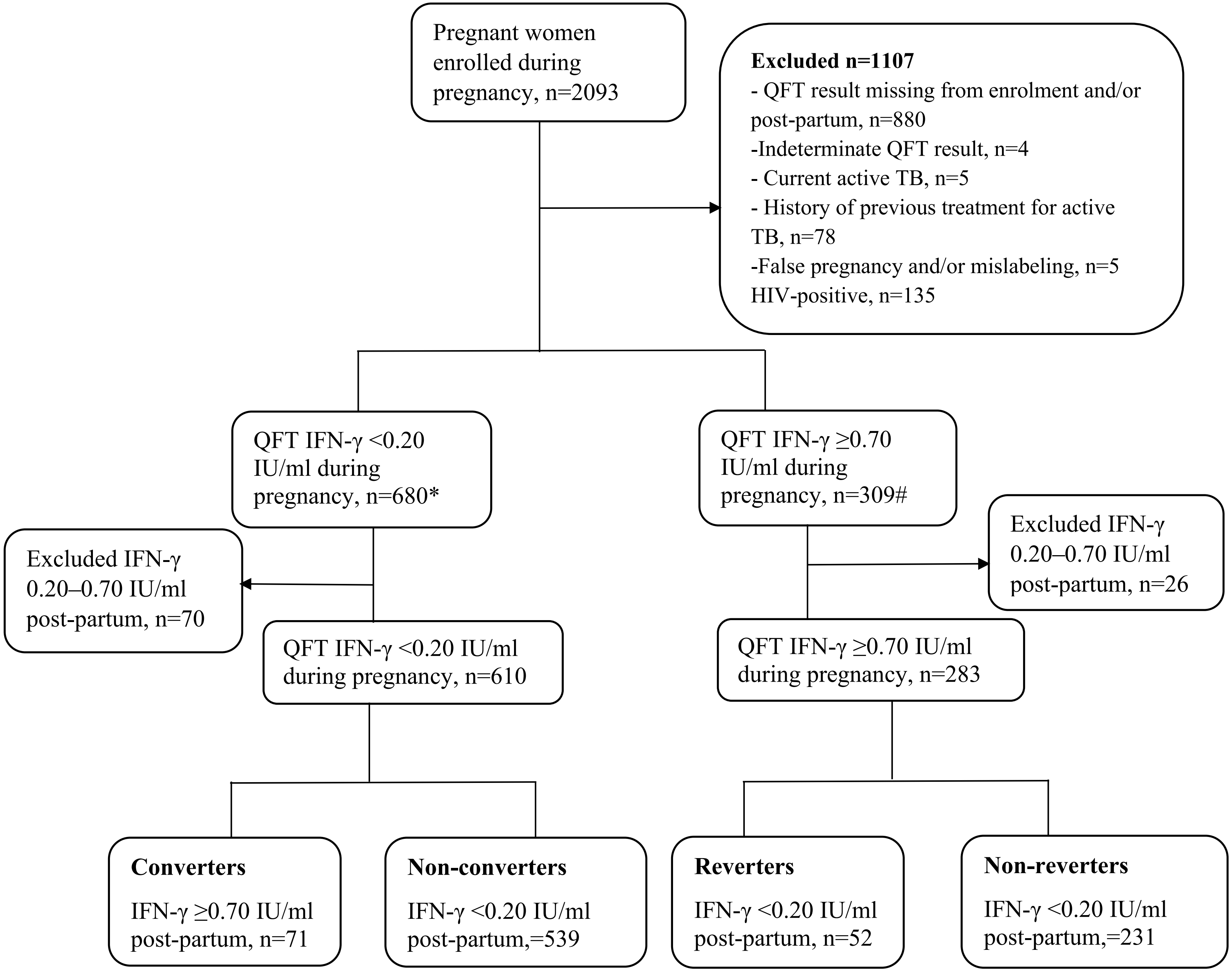
Pregnant women enrolled during pregnancy, n=2093
Excluded n=1107
- QFT result missing from enrolment and/or post-partum, n=880
-Indeterminate QFT result, n=4
- Current active TB, n=5
- History of previous treatment for active TB, n=78
-False pregnancy and/or mislabeling, n=5
HIV-positive, n=135
QFT IFN-γ <0.20 IU/ml during pregnancy, n=680*
QFT IFN-γ ≥0.70 IU/ml during pregnancy, n=309#
Excluded IFN-γ 0.20–0.70 IU/ml post-partum, n=70
Excluded IFN-γ 0.20–0.70 IU/ml post-partum, n=26
QFT IFN-γ <0.20 IU/ml during pregnancy, n=610
QFT IFN-γ ≥0.70 IU/ml during pregnancy, n=283
Converters
IFN-γ ≥0.70 IU/ml post-partum, n=71
Non-converters
IFN-γ <0.20 IU/ml post-partum,=539
Reverters
IFN-γ <0.20 IU/ml post-partum, n=52
Non-reverters
IFN-γ <0.20 IU/ml post-partum,=231

**Supplementary Figure 3** Flow chart of pregnant women included for analysis of QFT conversions and reversions between pregnancy and post-partum.

*IFN-$\gamma$ <0.20 IU/ml (in TB1 and TB2 antigens)

# IFN-$\gamma$ $\geq$0.70 IU/ml (in TB1 and/or TB2 antigen)

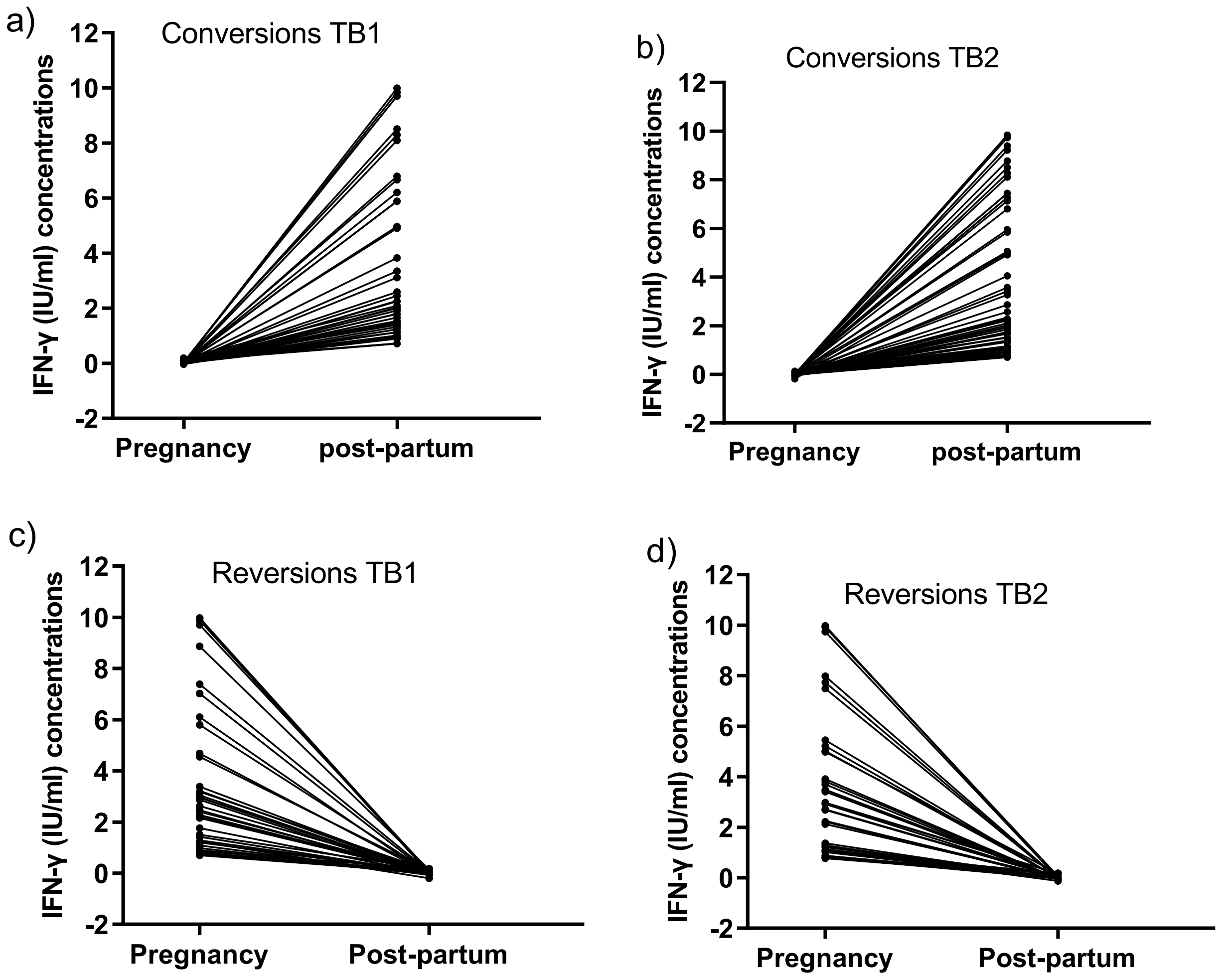
a)
Conversions TB1
IFN-γ (IU/ml) concentrations
12
10
8
6
4
2
0
-2
Pregnancy
post-partum
b)
Conversions TB2
IFN-γ (IU/ml) concentrations
12
10
8
6
4
2
0
-2
Pregnancy
post-partum
c)
Reversions TB1
IFN-γ (IU/ml) concentrations
12
10
8
6
4
2
0
-2
Pregnancy
Post-partum
d)
Reversions TB2
IFN-γ (IU/ml) concentrations
12
10
8
6
4
2
0
-2
Pregnancy
Post-partum

**Supplementary Figure 4** The line plots show participants with QFT IFN-γ conversions (n=71 [11.6%] among 610) and reversions (n=52 [18.4%] among 283) during pregnancy and post-partum in TB1 and/or TB2 antigen stimulation. Converters: IFN-γ <0.20 IU/ml in response to both TB antigens during pregnancy with IFN-γ ≥0.70 IU/ml at post-partum in response to TB1 (a) and TB2 (b) antigens. Reverters: IFN-γ ≥0.70 IU/ml in TB1 (c) and TB2 (d) antigens during pregnancy with IFN-γ<0.20 IU/ml at post-partum in response to both TB antigens.
